# TREATMENT OF PAIN DURING THE ACUTE CARE STAY FOR PATIENTS LIVING WITH DEMENTIA

**DOI:** 10.1093/geroni/igad104.0702

**Published:** 2023-12-21

**Authors:** Marie Boltz

**Affiliations:** Pennsylvania State University, State College, Pennsylvania, United States

## Abstract

The purpose of this study was to describe pain among hospitalized patients living with dementia, the use of pharmacologic and nonpharmacologic treatment, and to compare treatments among those with and without pain. Data from the first 365 participants in the FFC-AC-EIT study was used. The mean age of participants was 83 (SD=5), the majority was female (65%) and White (67%). Controlling for treatment, between admission to discharge there was a significant decrease in pain from 36% having pain on admission to 31% at discharge (F=5.30, p=.02). At discharge 125 (30%) had pain and 8 (6%) of individuals with pain received no nonpharmacological or pharmacological treatments during the hospital stay. The most frequently used pharmacological intervention was acetaminophen (52%), then tramadol (8%). Comfort measures (49%), physical activity (46%), and communication (38%) were the most common nonpharmacologic approaches. Between those with and without pain there was no difference in use of pharmacologic interventions (F = .38, p =.54) and there was less use of nonpharmacological treatments for those with pain (F=10.24, p=.002). The majority of patients living with dementia were treated for pain, but an ongoing focus is needed to assure optimal pain management for all patients. More attention to use of and evaluation of effectiveness of pharmacologic and nonpharmacologic treatments alone or together are needed for hospitalized patients living with dementia.

